# Nanoscale monitoring of the initial stage of water condensation on a printed circuit board

**DOI:** 10.1016/j.heliyon.2025.e42117

**Published:** 2025-01-21

**Authors:** Alekszej Romanenko, Ali Gharaibeh, Bálint Medgyes, Peter Petrik

**Affiliations:** a Doctoral School of Chemistry, Eötvös Loránd University, Pázmány Péter sétány 1/A, Budapest, H-1117, Hungary; b HUN-REN Centre for Energy Research, Konkoly-Thege út 29-33, Budapest, H-1121, Hungary; c Department of Electronics Technology, Faculty of Electrical Engineering and Informatics, Budapest University of Technology and Economics, Műegyetem rkp. 3, Budapest, H-1111, Hungary; d Department of Electrical Engineering, Institute of Physics, Faculty of Science and Technology, University of Debrecen, Bem tér 18, Debrecen, 4026, Hungary

**Keywords:** 0000, 1111, Electrochemical migration, Sn surface finish, Condensation, Ellipsometry, Water adsorption

## Abstract

Electrochemical migration is a critical factor contributing to failures in electronics due to humidity. When moisture accumulates on conductor-dielectric-conductor systems under bias voltage, electrochemical processes can be triggered, leading to the growth of metallic dendrites that may ultimately result in system failure. Despite its significance, many aspects of electrochemical migration remain unresolved, particularly regarding the physical characteristics of liquid buildup that facilitate dendrite growth and short circuit currents. While there are a few techniques that can measure water adsorption on the nanoscale, most conventional methods focus on water droplets within the size range of visible light wavelengths. In this study, we implemented a combined electrical-optical-ellipsometric measurement on FR-4 printed circuit boards featuring Sn surface finishes. Our experimental setup allowed for the measurement of water condensation across a wide range of thicknesses, while simultaneously monitoring the solder mask and metal electrodes during cooling. The ambient temperature of 25 ^∘^C and a relative humidity of 60% were constant during the measurement. By employing this approach, we elucidated the mechanisms of dendrite formation and short circuit currents, demonstrating that the water film remains continuous between droplets on the solder mask surface. Compared to Sn the nucleation was delayed on the solder mask with a larger surface coverage at smaller thicknesses. This comprehensive methodology provides crucial insights into the electrochemical migration process, enhancing our understanding of the underlying phenomena that contribute to electronic failures due to humidity. Our work highlighted the complementary nature of ellipsometry and optical imaging.

## Highlights

• Ellipsometry effectively monitored water condensation on an FR-4 board with Sn finish.
• Combined ellipsometry and electrical measurements tracked water adsorption on a PCB.
• Ellipsometry and optical imaging provided complementary insights into water adsorption.
• Continuous water films between droplets drove the dendrite formation.

## Graphical abstract

**Figure:**
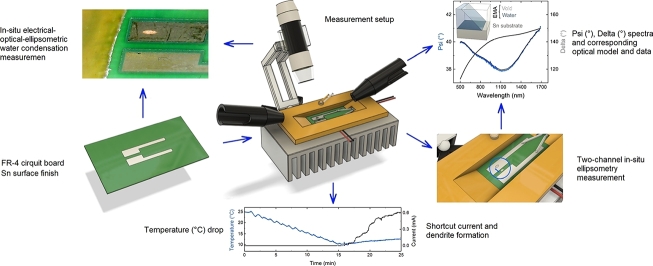

## 1 Introduction

Electrochemical migration (ECM), a humidity-induced failure mechanism, has emerged as a critical reliability issue due to the increasing use of miniaturized electronic systems. One key feature of ECM is the presence of humidity on conductor-dielectric-conductor systems under bias voltage, which facilitates ion migration [1], [2]. The ECM process progresses through four stages: formation of an electrolyte layer, dissolution of metal, ion migration, and finally, deposition of metal ions [1], [2]. This deposition process often leads to dendrite growth, which lowers the surface insulation resistance (SIR) between conductive components, eventually causing potential short circuit failure [3], [4]. Since the restriction on lead usage in electronics due to its toxicity, ECM has gained greater attention, especially in Pb-free alloys [5]. This shift has prompted an increased focus on the ECM behavior in Sn and Sn-based Pb-free solder alloys [1], [2], [6], [7].

Three primary methods are widely used to assess ECM susceptibility: the water drop (WD) test, thermal humidity bias (THB) test, and thin electrolyte layer (TEL) test [8]. The WD and TEL tests apply a predefined electrolyte volume, either as a droplet or a thin layer, directly between biased electrodes, which initiates the ion migration process more rapidly. In these tests, the time to failure (TTF) is generally shorter than in the THB test, which includes the formation of the electrolyte layer. However, the THB test offers a significant advantage in terms of control over critical variables like temperature, relative humidity, and bias voltage, making it more representative of real-world conditions. This test is often preferred in industrial settings and laboratory experiments where accuracy in simulating operational environments is crucial [9], [10].

Different test methods are suitable for investigating ECM in visible electrolytes, but only the THB test can be used to study ECM within invisible moisture films. Literature shows that not all metals exhibit ECM migration within these unseen moisture films [11], [12], [13], [14]. One of the main advantages of the THB test is that it provides continuous monitoring of the electrolyte layer formation throughout the ECM process, offering better insight into real operating conditions for electronic devices. The THB test results demonstrate that the mean time to condensation (MTTC) must be added to the mean time to dendrite formation (MTTD) to calculate the overall mean time to failure (MTTF). While MTTC depends on factors like surface roughness and the thermal properties of the materials involved, MTTD is influenced primarily by the electrolyte's pH, solubility, and surface finish characteristics [15], [16], [17].

Although the THB test provides substantial information on ECM, it lacks the ability to accurately measure the thickness of the electrolyte layer triggering ECM. For example, in the TEL test, the thinnest electrolyte layer measured was 30 microns, yet it is suggested that in real-world environments the electrolyte layer could be significantly thinner [18]. Consequently, details about the precise formation of electrolyte layers remain scarce in current literature. While WD and TEL tests are commonly used to evaluate ECM susceptibility of metals and Pb-free alloys, the THB test is often selected for studies that aim to more accurately simulate real-world operating conditions in electronic systems. This research focuses on investigating water accumulation at the nanometer scale during the ECM process on electroplated Sn surface finishes, utilizing ellipsometry as the primary tool for precise and accurate measurements.

Ellipsometry, a highly sensitive, non-destructive technique, is particularly suitable for detecting nanoscale changes in optical properties. It enables accurate, contact-free probing of a wide variety of materials, making it ideal for in-situ characterization and nanostructure analysis [19], [20], [21], [22]. In addition to ellipsometry [23], other surface-sensitive techniques [24] such as imaging [25], X-ray scattering [26], [27], nuclear magnetic resonance (NMR) [26], [27], Second Harmonic Generation (SHG) [28] and Sum Frequency Generation (SFG) [29] spectroscopies are available. These methods are widely used to study molecular arrangements and surface properties at interfaces, providing additional insights into processes like water condensation and ion migration at the molecular level [28], [29], [30]. However, for this study, ellipsometry remains the primary technique due to its ability to provide detailed thickness and refractive index information, ensuring highly accurate data collection throughout the ECM process.

Ellipsometry, when paired with theoretical models, allows researchers to extract material properties from the data with high accuracy, strengthening the reliability and depth of the study's findings. (Through the application of theoretical models, ellipsometry allows for the accurate extraction of material properties from collected data, enhancing the robustness and precision of the results [31], [32], [33].) Despite its frequent use in various fields, ellipsometry has seen limited application in studies of water condensation and ECM. This study seeks to address that gap by leveraging ellipsometry to investigate water accumulation at the nanometer scale during the ECM process.

## 2 Materials and methods

**Figure 1:**
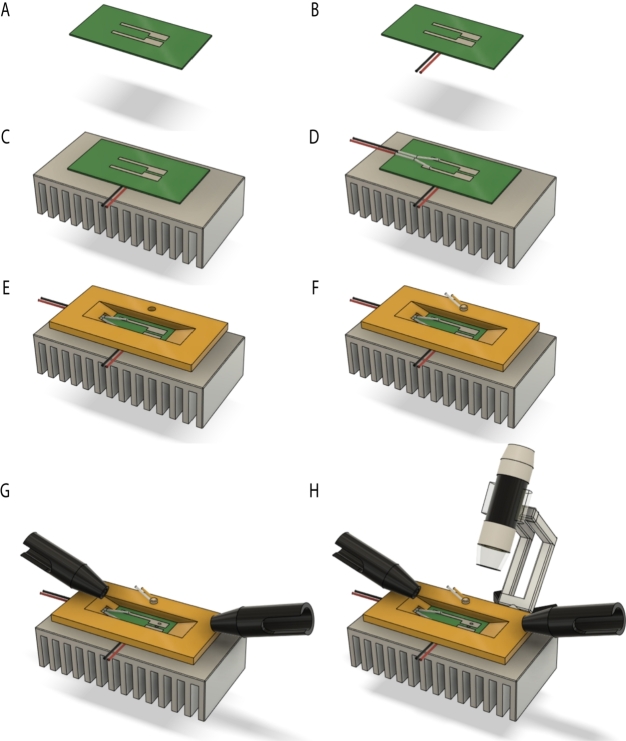
Step-by-step assembly process of the experimental setup, illustrating: (A) the layout of the sample, (B) the attachment of the thermoelectric Peltier cooling module, (C) the installation of the heat sink, (D) the soldering of wires for constant potential application, (E) the addition of protective and stabilizing covers, (F) the installation of the thermometer, (G) the adjustment of focusing probes for the spectroscopic ellipsometer, and (H) the integration of the microscope and camera system.

In this study, the test sample was an FR-4 printed circuit board (PCB) with electroplated tin (Sn) electrodes (Fig. 1A). The FR-4 board used is a glass-reinforced epoxy laminate material with flame-retardant properties, capable of withstanding temperatures up to 260–280 ^∘^C. The specific board was manufactured by UniPCB, Hungary, using subtractive printed wiring technology. The FR-4 surface was covered by an epoxy-based solder mask to protect the substrate. The solder mask is made of photosensitive ink. Two-component ink with epoxy resin base is applied, its usage is similar to photoresist technology. The ink is applied onto the surface of the substrate using screen printing method. In this case screen printing does not determine the pattern of the layer, only its thickness. Then it will be dried in air convection oven. After exposition and developing, the substrate will be burned in.

The electroplated Sn patterns on the PCB had dimensions of 2 mm × 5 mm, with a 0.5 mm gap between the conductor lines. The thickness of the electroplated Sn film was approximately 10–12 μm. This Sn layer was not made thicker due to its technological function in the PCB manufacturing process. Specifically, during wet etching, Sn acts as a metal mask for the removal of the underlying Cu layer. Depending on customer requirements, the Sn surface may remain as the final surface finish or be replaced by other finishes, such as immersion silver (Ag). The electroplated Sn has two roles in this technology: (i) acting as a metal mask during wet chemical etching of Cu; (ii) after etching, Sn acts as a surface finish protecting the Cu layer from oxidation and ensure a good (not excellent) surface wettability from the soldering point of view. In terms of composition, the Sn layer may include traces of Cu from the underlying layer, whereas the photosensitive ink is an epoxy resin (CAWN1346 and CAWN1274 from SunChemical – see the excerpt from the technical information leaflet in the supplementary document) revealing a complex structure involving monomers, hardening agent, and modifiers such as fillers, plasticizers, pigments, UV stabilizers, and other components the roles of which cannot be the aim for discussion in this study.

To achieve cooling of the sample, a Peltier thermoelectric module (TEC1-12715) was employed (Fig. 1B), particularly its upper surface. A heat sink (Fig. 1C) with effective thermal dissipation capability (≈4 W) was affixed to the Peltier module's warm side using thermal paste to maximize heat transfer. Similarly, the sample was affixed to the opposite side of the Peltier module (Fig. 1B, heat conduction rate of 13.5 W) using the same thermal paste to enhance heat conduction. The voltage applied to the Peltier module (Fig. 1D) was gradually adjusted in the 0 - 5 V range using a Keysight E3632A 120W Power Supply while measuring the temperature on the sample using a thermocouple. The temperature was not controlled, only the voltage of the Peltier module was changed, because the measurement was quick enough to follow the drop formation. We didn't measure the heat conduction between the elements (the heat conduction rate of FR-4 and the Peltier module is estimated to be approx. 4 W, the heat gain from the environment 0.1 W), because the cooling power and air contribution was sufficient to produce the effect in a way that could be monitored by the applied methods. The structure will later be modeled by finite element method to optimize the cooling.

Data collection, including the current between the two Sn films (Fig. 1D), the voltage across the Peltier module (Fig. 1E), and sample temperature on the surface (Fig. 1F), was performed using the NI DAQExpress software and the NI USB-6212 Multifunction I/O Device. A potential of 10 V was also applied between the two Sn electrodes (Fig. 1D). The temperature and relative humidity of the ambient around the setup were controlled at 25 ^∘^C and 60%, respectively.

The power limit of the heat sink was reached after approximately 16.7 minutes of operation. The cooling process was specifically designed to reduce the intensity of a targeted phenomenon until a short circuit current was detected. This short circuit current serves as an indicator of a significant change in the system's behavior, suggesting that the cooling mechanism was sufficiently effective to achieve the desired outcome. Further investigation into the control mechanisms governing the cooling process beyond this threshold was considered unnecessary, as the experiment successfully met the established criteria for the intended effect.

Contact angle measurements were performed on Sn surfaces of the test printed circuit boards (PCBs) using a custom-built contact angle goniometer. The measurements were conducted in a near-saturated water vapor environment (relative humidity, RH ≥ 85%) at a temperature of 24 ^∘^C. Ultrapure water, produced by a Sartorius Arium Mini ultrapure water system (resistivity: 18.2 MΩ⋅cm, surface tension, $\gamma_{\text{water}}=72.25$ mN/m at 24 ^∘^C), was used as the test liquid. The samples were measured in their as-received condition, without any additional surface cleaning. Advancing contact angles were determined by the sessile drop method using the drop build-up technique, with the long and narrow Sn surface oriented perpendicular to the optical axis. The measurements were analyzed using ellipse fitting for contact angle evaluation.

**Figure 2:**
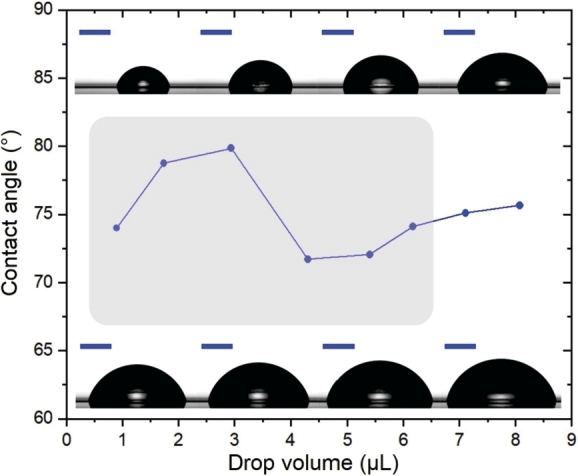
Advancing water contact angles were measured as a function of sessile drop volume on the tin surface of the test PCB. Insets present images of droplets with volumes ranging from 1 to 8 μL, with a blue scale bar of 1 mm. The drop size effect is evident within the shaded region.

During the advancing contact angle measurements, the volume of the sessile drop was incrementally increased in 1 μL steps. To minimize the influence of drop size on contact angle measurements, as previously observed on surfaces with very small contact diameters (even on close-to-ideal surfaces [34]), only contact angles measured for drops exceeding 7 μL were considered. For larger drops, the contact angle remained stable with further volume increase. Due to the narrowness of the Sn surfaces, part of the contact line intersected with the plastic surface at this drop volume. However, the analyzed region of the triple line was confined to the Sn surface, with the Sn surface consistently aligned perpendicular to the optical axis. This necessitated the use of elliptical fitting for evaluation. Although the Young–Laplace fit provided similar results, the difference between the two methods remained below 0.5^∘^. The resulting advancing water contact angle was $75.6^{\circ }\pm 0.6^{\circ }$ (Fig. 2).

A Woollam M-2000DI rotating compensator spectroscopic ellipsometer has been used (Fig. 1G) to determine the ratio between $r_{p}$ and $r_{s}$, which represent the complex reflection coefficients of light polarized parallel and perpendicular to the plane of incidence, respectively. The Ψ (the amplitude ratio upon reflection) and Δ (the phase shift) spectra were acquired simultaneously for 700 spectral points using [35]

(1) $$\frac{r_{p}}{r_{s}}=\tan(\Psi )\cdot e^{i\Delta }.$$

This measurement covered a wavelength range of 193 to 1690 nm with a temporal resolution of one second per whole spectra and a thickness resolution of less than 1 nm. We employed focusing probes to achieve a smaller and more intense light interrogation area on the surface, enabling measurements between the two Sn planes on the solder mask surface. Ultimately, the size of the light spot achieved was ≈ 0.3 mm × 0.9 mm. Subsequently, the acquired measurement data were processed and analyzed using the CompleteEASE 6.42 software. The ECM mechanisms were also in-situ observed using a USB microscope (type: XCAM MAN1001-SA) for visual inspection (Fig. 1H).

**Figure 3:**
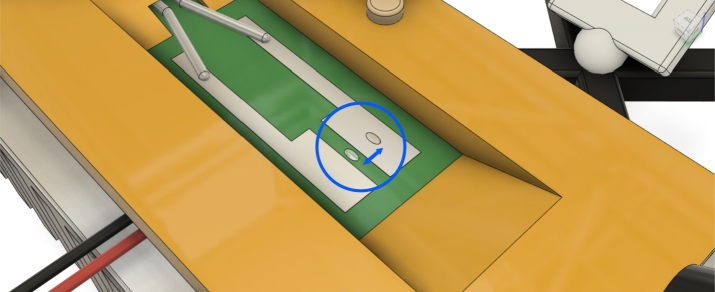
Illustration of the two-channel simultaneous in-situ measurement, where the focused light spot is alternately positioned between the Sn film and the FR-4 board to examine both surfaces under identical conditions [36].

During the measurements, the sample was systematically shifted relative to the light beam, ensuring that the focused light spot alternated between two specific positions: directly over the center of one of the Sn films and the gap between them on the surface of the solder mask (Fig. 3). This method enabled the investigation of both surfaces under identical experimental conditions, such as temperature, humidity, and cooling.

The angle of incidence was set to 70^∘^, chosen as an optimal balance between minimizing the spot size and maintaining adequate light intensity. This angle is also close to the maximum achievable angle for precise focusing during the measurement. The measurement time at each position was 6 seconds, with the motor requiring 0.6 seconds to precisely move the stage to the next point. Thus, each measurement point was revisited every 13.2 seconds, with a 6-second data acquisition window. This cycle time was carefully selected to strike a balance between minimizing noise and capturing the kinetics of the process, ensuring that the temporal resolution was sufficient to monitor the ongoing changes during the measurement. Although the default measurement uncertainty of both Ψ and Δ is 0.01^∘^, the uncertainty that is translated to the determined quantitative model parameters depends on the noise and parameter correlations. Therefore, the error bars are plotted and discussed for the fitted parameters, as shown in the next section.

Since local environmental conditions can significantly influence the water condensation process, temperature and humidity were monitored continuously in real-time throughout the experiment by MAX-MIN Thermo Hygro (the MAX-MIN Thermo-Hygrometer was positioned at a distance of 20 cm from the measurement setup). Also an air conditioning system was employed to stabilize these conditions, ensuring that any fluctuations were minimized. By maintaining controlled environmental parameters, systematic errors were eliminated, and the repeatability of the experiments was enhanced. However, it has to be noted that laboratory climatization alone does not provide a fully controlled environment with respect to humidity and temperature. Since our study does not correlate water adsorption directly with environmental conditions, our primary aim was to induce water adsorption/condensation conditions, even if in an uncontrolled manner. We acknowledge that the open nature of our system, even with air conditioning, does not allow for precise control over temperature and humidity throughout the entire experiment. However, the primary objective was to create conditions conducive to water adsorption, focusing not on strict environmental control but on achieving comparable adsorption conditions across different measurement methods. Consequently, we did not aim to correlate adsorption behavior with exact environmental parameters, as this was beyond the scope of our study. Instead, the data comparisons were made under consistent conditions across all tests, ensuring that observed trends in electrochemical migration (ECM) reflect the measurements themselves rather than fluctuations in humidity or temperature. The focus of our correlation was on the values measured across different methods, under the same conditions.

This approach allowed for a direct comparison between the two surfaces (Sn films and solder mask), ensuring that any observed differences in the processes could be attributed solely to the surface properties, as all other variables were kept constant.

## 3 Results and discussion

**Figure 4:**
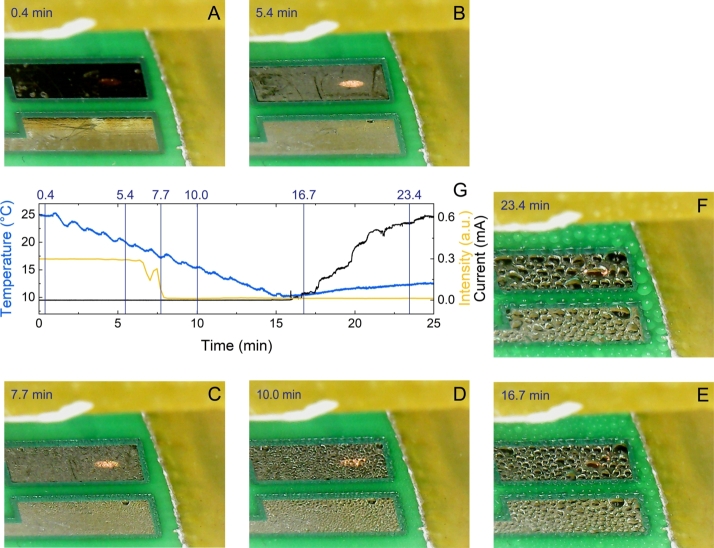
Progression of water condensation on the electroplated Sn surface and the solder mask on an FR-4 substrate during cooling. At approximately 16.7 minutes, the Peltier module reached its thermal capacity limit, leading to a gradual increase in temperature. The yellow curve represents the intensity of light reflected from the Sn layer, as recorded by ellipsometry, in arbitrary units. This curve correlates with the cooling process and subsequent heating, illustrating changes in the surface properties over time.

The water condensation and adsorption stages were studied on an FR-4 printed circuit board using a combination of two-channel in-situ ellipsometry [36] (Fig. 3) and optical imaging [25] (Fig. 4). Multiple experiments were conducted under identical conditions—relative humidity of 60%, ambient temperature of 25 ^∘^C, and a fixed bias voltage of 10 V—with current monitored throughout the process. The results showed a high level of consistency. Importantly, the back-and-forth shifting of the sample during the two-channel measurement had no significant impact on droplet formation or movement, which was confirmed by both the ellipsometric baselines and optical camera observations.

Different stages of condensation and adsorption were separated from the measurements. In the early stage of the adsorption process, the size of the droplets was not large enough to scatter light and make the objects visible to the camera (Fig. 4A). However, after approximately 5.4 minutes of the adsorption process (Fig. 4B), the size of the droplets became comparable to be seen by the optical camera. The increase of visibility by optical imaging is caused by the growing droplets reaching a size comparable to the illumination wavelength (Fig. 4C, D). This was accompanied by a notable decline in intensity value extracted via ellipsometry between 5.4 to 7.7 minutes of the adsorption process as a result of increased light scattering caused by the rapidly increasing density of water droplets (as shown in Fig. 4B and 4C), a declining trend indicated by the yellow curve in Fig. 4G. At the 16.7-minute mark, a short circuit current was observed (Fig. 4G), indicating the formation of large droplets on the Sn surface (Fig. 4E), which continued to grow with further condensation (Fig. 4F).

The current was derived by monitoring real-time, in-situ changes in the voltage applied to the system. This approach allowed for accurate tracking of current variations throughout the experiment. Detailed information on the test platform and experimental procedure can be found in Ref. [37]. The chemical composition of the formed dendrites is primarily dominated by Sn, with trace amounts of Cu potentially present beneath the Sn surface finish; however, Cu is not the dominant element. It is noteworthy that, in certain instances, the current manifested in the form of peaks, rather than the steady escalation depicted in Fig. 4G. This variance can be attributed to the current's potential to damage dendrites associated with the short circuit.

The interaction of these Sn dendrites with the components of the conductor-dielectric-conductor system, consisting of Sn/FR-4/Sn, can lead to the formation of short circuits, as previously discussed. Additionally, it has been observed that under certain conditions, dendrites may “disappear” due to relatively high current densities, which induce evaporation. While this phenomenon may resemble a self-healing process, it is misleading. The underlying cause is the carbonization of the FR-4 substrate, resulting in an irreversible drop in resistance.

**Figure 5:**
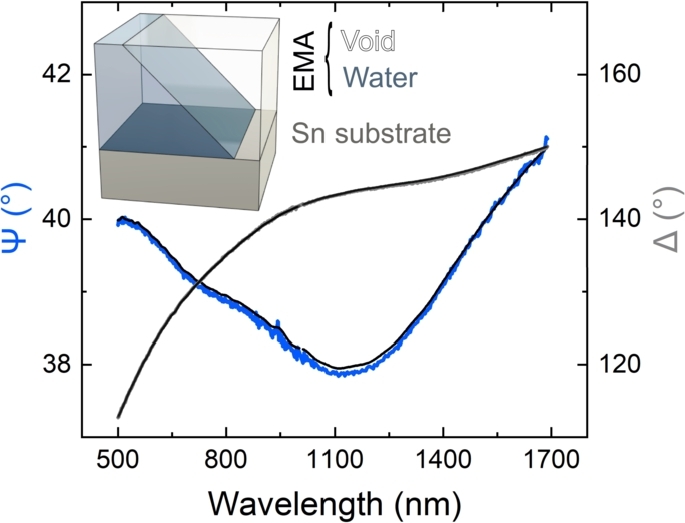
Typical measured (light gray and light blue lines) and fitted (black lines) ellipsometry spectra on the Sn film of the sample at 7 minutes. The inset shows the optical model used for the evaluation. The dielectric function of Sn was determined prior to the measurement, taking into account the presence of native oxide.

The ellipsometry data were analyzed using an optical model that included a surface layer representing the adsorbed water molecules and an Sn substrate modeled with Lorentz oscillators [38] (Fig. 5). The surface layer was represented as a composition of water and air, calculated using the Bruggeman effective medium approximation (EMA) [39], [40], [41]. At each time point, the thickness (*d*, in units of nm) and volume fraction of water ($f_{w}$, with 1 representing 100% and 0 representing 0%) were fitted simultaneously.

**Figure 6:**
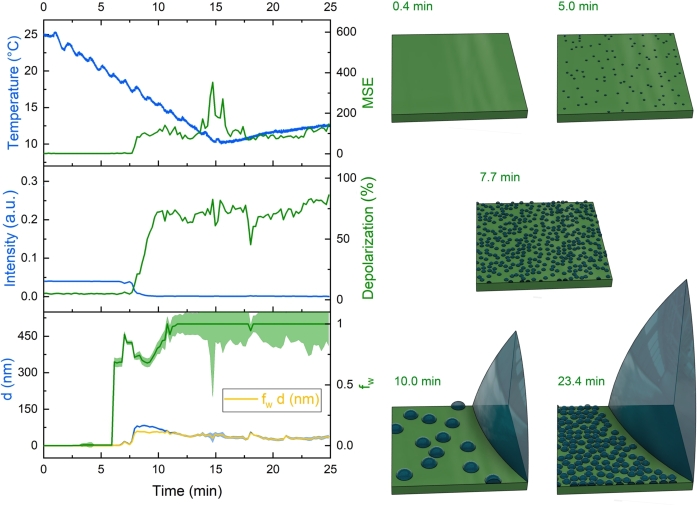
Measurements of raw parameters (light intensity and depolarization of light at a wavelength of 632.9 nm) and derived parameters (thickness *d* and volume fraction *f*_*w*_) acquired via ellipsometry on the solder mask portion of the sample during controlled cooling (refer to the temperature graph). The mean square error (MSE) indicates the fit quality. A schematic diagram illustrating the assumed growth of water condensate at specified time points is provided on the right. The cone-like slices represent the dimensions of the largest water droplets in comparison to the smaller ones. The depicted water droplets reflect their average sizes at each time frame, as the standard deviation of droplet sizes cannot be determined from the measurements.

**Figure 7:**
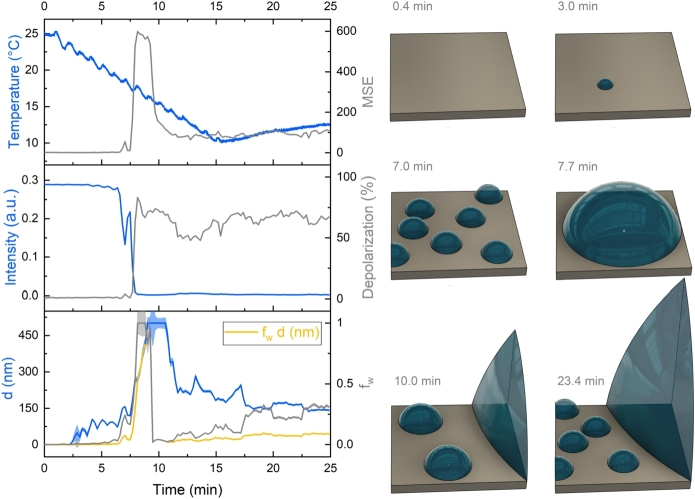
Measurements of raw parameters (light intensity and depolarization of light at a wavelength of 632.9 nm) and derived parameters thickness *d* and volume fraction *f*_*w*_) acquired via ellipsometry on the Sn layer during the controlled cooling of the sample (refer to the temperature graph). The mean square error (MSE) indicates the fit quality. A schematic diagram illustrating the assumed growth of water condensate at specified time points is provided on the right.

The dielectric function of the Sn substrate was determined from the measured spectra prior to water adsorption and was held constant throughout the rest of the process. The quality of the fit between the calculated and measured spectra was evaluated using the mean square error (MSE), defined as the weighted sum of squared differences between the calculated and measured Ψ and Δ angles (in degrees) across all wavelengths. Lower MSE values indicate a better fit quality. The raw error in measuring the Ψ and Δ ellipsometry angles was approximately 0.1^∘^. Uncertainty in the volume fraction parameter ($f_{w}$) is represented as ‘error stripes’ in Figure 6, Figure 7, as error bars were not used due to the large volume of in-situ measured data. These uncertainties are particularly pronounced for $f_{w}$, which is sensitive to correlations when fitting the optical model to the raw spectra. This reflects how ellipsometry can be considered a ‘quantitative spectroscopy,’ as it not only measures the raw spectra but also enables the construction of a quantitative model that includes layer thicknesses and component volume fractions. Although the uncertainty and detection limits are minimal (less than the width of the lines in the graphs), they are more apparent in the magnified scale shown in Figs. S1 and S2 in the supplementary material. Due to high scattering and low intensity at short wavelengths, only the range from 500 to 1690 nm was used for the evaluation [38].

It is important to note that the droplet size can be determined from the fitted thickness of the layer composed of droplet and air. In this model the effective refractive index of this layer can be calculated using the volume fraction of water as a parameter, and the thickness of the layer is equivalent with the size of the droplets [42]. The growth rate was determined by calculating the gradient of the layer thickness (*d*). The thickness, as determined by ellipsometry, reflects the average size of the largest visible droplets within the measured area. This comprehensive approach ensures the reliability of the extracted parameters, enhancing the overall accuracy of the analysis.

The evaluation of the ellipsometry spectra measured on the surface of the board between the electrodes and the Sn layer is shown in Figure 6, Figure 7, respectively. Both measurements were executed concurrently, ensuring synchronized timing as indicated by the aligned horizontal axes (a similar approach was used in Ref. 36). Additionally, the vertical axis values are standardized across both graphs for enhanced visual comparison. The MSE values, indicative of the fit quality [43], serve as crucial signatures for assessing the accuracy of the employed optical model at various stages of water adsorption.

The scattering of light due to the growing droplets on the mask area between the electrodes caused a decrease in intensity and a simultaneous increase in scattering-based optical depolarization (Figure 6, Figure 7). The optical depolarization can be measured by a rotating compensator ellipsometer such as the Woollam M-2000DI used in this study [44]. It can be measured because the rotating compensator ellipsometry can determine 12 of the 16 Muller matrix elements that include information on the depolarization of light, i.e., the fraction of polarization lost by reflection, since the sample is illuminated by a completely polarized light of known state of polarization [35]. Besides the scattering of light, the inhomogeneity in layer thickness also contributed to this optical depolarization [44].

Since ellipsometry is a ‘quantitative spectroscopy’, it not only measures the spectral response of the sample, but quantitative parameters can also be determined using an optical model [35], [23], [43]. A significant part of the model is the description of the spectral dependence of the optical properties of materials, i.e., the dispersion [45]. These are either tabulated data such as those used for Sn or polymers, but the dispersion of composite materials can also be determined using the effective medium theory [46], [47], [48], utilizing the combination of tabulated data determining the volume fractions. This way, the most significant quantitative parameters determined in our case by ellipsometry are the thickness (*d*) and the water volume fraction ($f_{w}$) in the surface layer [35], [23]. To measure these, we need to fit the whole spectral range using these parameters. This means that the spectral data are not independent from the modeling point of view, although the selection of the spectral range can naturally influence the results for a number of reasons described elsewhere [49], [50].

While there exists some correlation between the parameters, spectroscopic ellipsometry enables their independent determination owing to the minimal dispersion of water within the investigated spectral range, whereas the thickness *d* remains non-dispersive. Furthermore, the optical thickness ($f_{w}\cdot d$) is depicted as a yellow line, reflecting a value that is proportional to the quantity of water accumulated through condensation, thereby exhibiting reduced sensitivity to parameter correlation [51]. This approach has been previously applied and validated for organic materials in water [52], [36].

In general, adsorption occurs first when water molecules interact with the surface of Sn, which is followed by condensation. This happens because of the attractive forces between the surface and the water molecules, such as van der Waals forces, hydrogen bonding or electrostatic interactions. During adsorption individual water molecules are attracted and stick to the surface in a thin layer. Once a sufficient number of water molecules are adsorbed to the surface and if the local conditions (like temperature and humidity) are right, condensation can occur [25]. This involves the clustering of water molecules into larger droplets or liquid films, forming when the adsorbed layer reaches a critical thickness or when more water molecules come in from the environment and coalesce.

The first significant change detectable on the solder mask surface is a rapid increase of $f_{w}$ at the time of ≈ 6 minutes (temperature of 19 ^∘^C; see also a zoomed version in Fig. S1). Note that at this time frame, there is no drop of intensity, no increase of depolarization and MSE, showing that the evaluation is still in the most reliable range of the parameters. The increase in thickness is a few nanometers. The $f_{w}$ quickly reaches a value close to one, showing that the water molecules cover the surface of the solder mask uniformly in the initial stage of the process. The *d* remains a few nm for the next two minutes when it increases and quickly reaches ≈ 80 nm (at approximately 7.5 minutes). Simultaneously, the intensity drops and the depolarization increases, revealing the formation of droplets. The value of *d* and the high level of $f_{w}$ remains nearly constant for the rest of the process, showing that there is a few times 10 nm continuous water layer on the surface of the solder mask between the large droplets (see the schematic diagrams on the right-hand side of Fig. 6). The information about the average droplet size is reliable up to a thickness of approximately 500 nm. Beyond this threshold, data from larger droplets become inaccessible, and only information about the smaller droplets and the surface regions between the larger droplets can be accurately obtained.

The simultaneous monitoring of the Sn surface reveals different processes starting at ≈ 2.5 minutes (temperature of 22.5 ^∘^C) with an increasing *d* and a low $f_{w}$ (Fig. S2), which shows the formation of low-density, small-size (≈ 100 nm) droplets. Both *d* and $f_{w}$ increase rapidly at ≈ 6 minutes, which can be attributed to the formation of droplets, explained by the simultaneous drop in intensity and increase in depolarization. Shortly after this period, $f_{w}$ (from ≈9 min) and *d* (from ≈11 min) decrease. At this process stage most of the light is scattered from the droplets, and only the parts of the Sn surface between the droplets are visible for ellipsometry. The substantial drop of $f_{w}$ in this stage means that the water film is also non-continuous in the areas between the droplets, containing smaller droplets or islands with *d* in the range of 150-200 nm, as shown by the blue curve in Fig. 7 in the temporal region after ≈ 13 minutes.

The activities in the investigation of water condensation have gained remarkable momentum in the past decade, primarily due to its significance in material degradation and the development of surfaces for sensing and many other applications [24], [53], [54], [55], [56]. So far, the investigation of droplet formation has been restricted to the process stage, in which the droplets are large enough to be visible by optical microscopy [57]. The theory [24], process [53], dependence on relative humidity [57] and wettability [54], [58] have all been investigated in this regime, which corresponds to the time periods of our investigation after the reduction of intensity at time points of ≈7.5 and ≈7 minutes for the solder mask and the Sn surface, respectively.

In spectroscopic ellipsometry (SE), the primary methods of modeling the structure are combining the transfer matrix method for stratified layered structures and analytical parametric line shapes or the EMA for the dispersion within the layers. These approaches require that the sizes of structures and phases (*D*) are smaller than the wavelength (*λ*). The λ/D ratio of this so-called quasi-static limit is somewhere between 3 and 10 [46], [59], [42], [50]. In our case, the feature size *D* corresponds to the diameter of the forming droplets. Considering the wavelength range the size of a few hundred nanometers causes scattering and loss of intensity. These moments are characterized by a rapid intensity drop in Figure 6, Figure 7. The droplets of this size and above scatter the light that does not enter the detector arm of the ellipsometer. However, a portion of this scattered light comes to the detector, causing depolarization. This is why the depolarization increases simultaneously with the intensity decrease in both cases of the solder mask (Fig. 6) and the Sn (Fig. 7) substrates.

A remarkable feature that can be explained by the gradual change of the quasi-static limit is the simultaneous increase of *d* and MSE for the Sn substrate between ≈7 and 9 minutes (Fig. 7). This finding may be explained by the droplets growing in a size range that gradually invalidates an increasing part of the spectrum (EMA not valid due to scattering, the influenced spectral range being a function of the size) used for the fit that results in the increasing MSE. The subsequent drop in MSE is probably caused by the fact that the growing droplets are not visible anymore, and this way, they only contribute to the depolarization and not to the MSE. The drop of MSE cannot be observed on the solder mask due to the smaller sizes of the droplet that remain visible for SE after the decrease of the intensity (see the time range from ≈8 minutes in Fig. 6 with *d* of a few times 10 nanometers and $f_{w}\approx 1$).

Ellipsometry offers a distinct advantage in characterizing the transition region marked by the initial change in the signal (refer to the raw signal recorded at a wavelength of 632.9 nm in Fig. S3), particularly during the formation of the first optically visible droplets, where conventional techniques often fall short. Many prevalent methods, such as electron microscopy and X-ray photoelectron spectroscopy, require a vacuum environment and lack the capability to detect and monitor the evolution of molecular films with nanometer-level sensitivity. It is important to note that the method is designed to measure only the average value of the water layer thickness. It provides a representative measurement of the optical properties across the entire measured surface area.

The most important feature of SE is the separation between $f_{w}$ and *d*. Because the increased effective refractive index (which is proportional to $f_{w}$) and *d* change the measured Ψ and Δ ellipsometry angles identically, the separation is only available for spectroscopic ellipsometry. Once $f_{w}$ and *d* are separated, the droplet size and the coverage can be followed separately in the size range of nanometers. In our case, there is a characteristic difference in the behavior of *d* vs $f_{w}$ between the solder mask and Sn substrates in the above-mentioned initial pre-drop formation time range of ≈2.5 to 7.5 minutes. The fit results suggest a more significant initial drop size, a smaller density, and a significantly higher on-set temperature for the Sn substrate.

## 4 Conclusions

Two-channel in-situ ellipsometry was combined with electrical and optical imaging measurements. Ellipsometry was used to monitor water condensation on a solder mask-covered FR-4 board and an Sn surface finish simultaneously, while also monitoring the short circuit current and the optical image of the system. The initial stage of sub-nanometer level water adsorption was measured and interpreted for the first time on a printed circuit board by ellipsometry. The demonstration highlighted the complementary nature of ellipsometry and optical imaging. Ellipsometry provides the most significant intensity and sensitivity during the adsorption stage, which is invisible to optical microscopy. Conversely, optical microscopy offers more information during the other stage of adsorption. This approach enables the gathering of continuous and precise information regarding the surface's evolution. The short circuit current can be explained by the experimental results showing that the water film is continuous between the droplets on the solder mask surface, whereas it forms nanoislands on the Sn. The combined in-situ electrical-optical-ellipsometric measurement was shown to be a powerful tool in the high-sensitivity real-time study of electrochemical migration processes.

## CRediT authorship contribution statement

**Alekszej Romanenko:** Writing – review & editing, Writing – original draft, Visualization, Methodology, Investigation, Formal analysis, Data curation, Conceptualization. **Ali Gharaibeh:** Writing – review & editing, Writing – original draft, Methodology, Investigation, Formal analysis, Data curation. **Bálint Medgyes:** Writing – review & editing, Writing – original draft, Validation, Supervision, Resources, Project administration, Methodology, Investigation, Funding acquisition, Formal analysis, Data curation, Conceptualization. **Peter Petrik:** Writing – review & editing, Writing – original draft, Validation, Supervision, Resources, Project administration, Methodology, Investigation, Funding acquisition, Formal analysis, Data curation, Conceptualization.

## Declaration of Competing Interest

The authors declare that they have no known competing financial interests or personal relationships that could have appeared to influence the work reported in this paper.

## Data Availability

The data presented in this study are available on request from the corresponding author.

## Appendix A Supplementary material

MMC The supplementary material provides additional information on the determined values by the contact angle measurement, detailed figures on the in-situ ellipsometry measurements focusing on the initial stage of the process, the raw real-time ellipsometry data, as well as an excerpt of the technical information on the epoxy resin used as a solder mask. MMC
