# Supplementary material for: Nanoscale monitoring of the initial stage of water condensation on a printed circuit board
Source: Heliyon. 2025 Jan 21;11(2):e42117. doi: 10.1016/j.heliyon.2025.e42117 (PMC11795051; doi:10.1016/j.heliyon.2025.e42117)
Supplement: MMC — The supplementary material provides additional information on the determined values by the contact angle measurement, detailed figures on the in-situ ellipsometry measurements focusing on the initial stage of the process, the raw real-time ellipsometry data, as well as an excerpt of the technical information on the epoxy resin used as a solder mask. [file mmc1.pdf]

Nanoscale monitoring of the initial stage of water condensation on a printed circuit board

Alekszej Romanenko[1,1], Ali Gharaibeh[1], Bálint Medgyes[1], Peter Petrik[1,1,]

[a]*Doctoral School of Chemistry, Eötvös Loránd University, Pázmány Péter sétány 1/A, Budapest, H-1117, Hungary*
[b]*Centre for Energy Research, Konkoly-Thege út 29-33, Budapest, H-1121, Hungary*
[c]*Department of Electronics Technology, Faculty of Electrical Engineering and Informatics, Budapest University of Technology and Economics, Műegyetem rkp. 3, Budapest, H-1111, Hungary*
[d]*Department of Electrical Engineering, Institute of Physics, Faculty of Science and Technology, University of Debrecen, Bem tér 18, Debrecen, 4026, Hungary*

Mean CA (°)	Left CA (°)	Right CA (°)	Volume (μL)	Base Diameter (mm)
74.0	73.9	74.1	0.9	1.7
78.75	78.2	79.3	1.7	2.1
79.85	79.7	80.0	2.9	2.5
71.7	71.6	71.8	4.3	3.0
72.05	72.2	71.9	5.4	3.2
74.1	74.4	73.8	6.2	3.3
75.1	75.3	74.9	7.1	3.4
75.65	76.0	75.3	8.1	3.6

Table 1: The measured data correspond to water drops placed on the tin surface of the test PCB. The contact angle (CA), volume, and base diameter of each droplet were determined based on the size calibration of the images.

*Corresponding author. Email address: petrik.peter@ek.hun-ren.hu

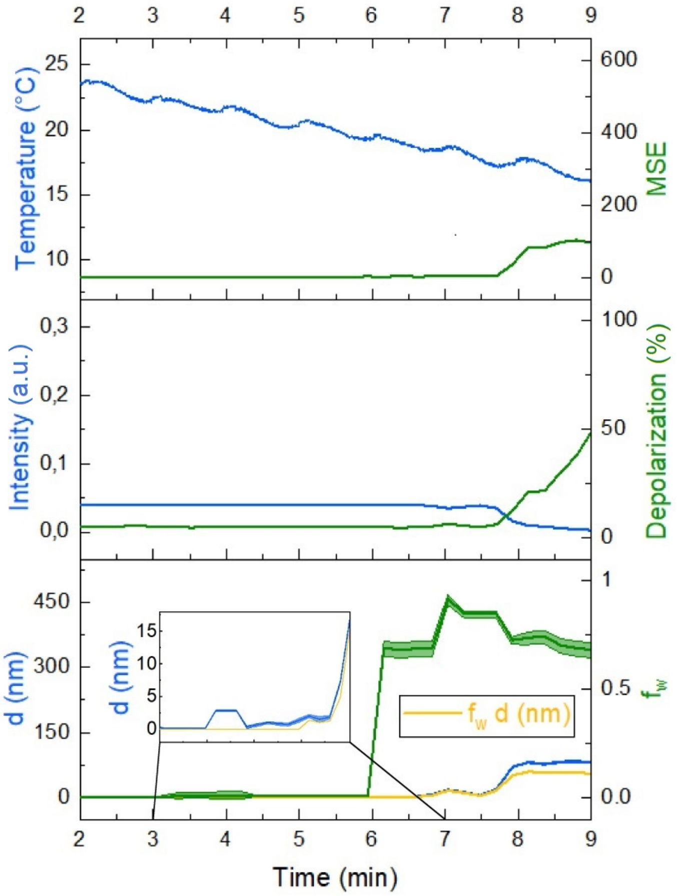

Figure S1: Raw (intensity, depolarization (at 632.9 nm wavelength)) and derived (d and f_w) parameters measured by ellipsometry on the resin part of the sample during the controlled cooling of the sample (see the temperature graph). MSE denotes the error of fit. The schematic diagram of the assumed growth of water condensate at the given moments is shown on the right-hand side of the figure.

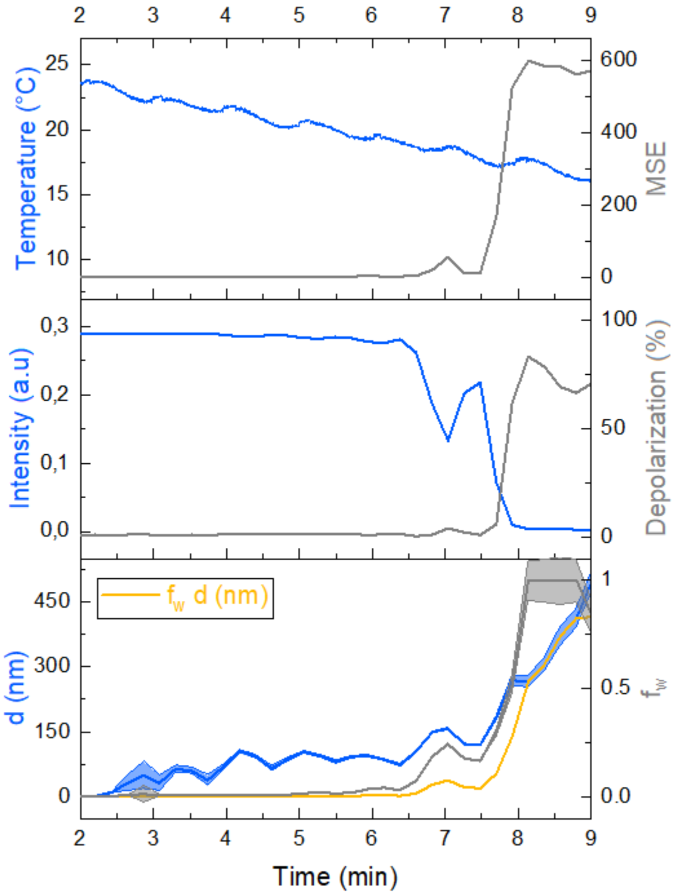

Figure S2: Raw (intensity, depolarization (at 632.9 nm wavelength)) and derived (d and f_w) parameters measured by ellipsometry on the Sn layer during the controlled cooling of the sample (see the temperature graph), MSE denotes the error of fit. Schematic diagram of the assumed growth of water condensate at the given moments.

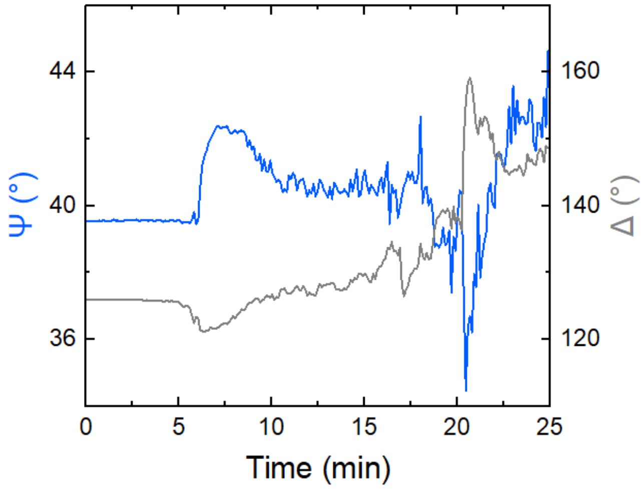

Figure S3: Measured ellipsometric angels Ψ and Δ at 632.9 nm wavelength on the Sn layer during the controlled cooling.

PHYSICAL PROPERTIES OF IMAGECURE® XV501T SCREEN

Component	Viscosity (Haake VT550)	S.G.	Flash point	Non Volatile Content
CAWN1318 / 1348	17.5 - 20.5 PaS.	1.22	> 70°C (158°F)	71.0%
CAWN1350	18.0 - 20.0 PaS.	1.22	> 70°C (158°F)	71.0%
CAWN / CAWP1346	17.5 - 20.5 PaS.	1.24	> 70°C (158°F)	71.0%
CAWN2004	17.5 - 20.5 PaS.	1.22	> 70°C (158°F)	71.0%
CAWN1375	17.5 - 20.5 PaS.	1.22	> 70°C (158°F)	71.0%
CAWN2117	17.5 - 20.5 PaS.	1.22	> 70°C (158°F)	69.5%
CAWN1272 / 1274 / 1347	28.0 - 34.0 PaS.	1.39	> 70°C (158°F)	78.0%
**Volatile Organic Content (VOC)**				360 - 400g./L.

PHYSICAL & CHEMICAL PROPERTIES OF IMAGECURE® XV501T CURED FILM

**Solder Resistance**	MIL-PRF-55110F<br>IPC SM840C	30 secs @ 288°C (550°F)<br>10 secs @ 260°C (500°F)
**Resistance to Solder Levelling**		> 5 passes
**Resistance to Fluxes**	IPC SM840C	Pass
**Electroless Ni/Au Plating**		Pass
**Hydrolytic Stability**	IPC SM840C Class H	Pass
**Solvent, Cleaning Agent, & Flux Resistance**	IPC SM840C Class H	Pass
**Fungal Resistance**	IPC SM840C Class H	Pass
**Thermal Shock**	IPC SM840C Class H<br>MIL-PRF-55110F<br>MIL-STD-202G	Pass<br>Pass<br>Pass
**Chemical Resistance**	IPA<br>1,1,1 Trichloroethane<br>MEK<br>Methylene Chloride<br>Alkaline Detergent<br>Fluxes	>1 hour<br>>1 hour<br>>1 hour<br>>1 hour<br>>1 hour<br>>1 hour
**Abrasion Pencil Hardness**	IPC SM840C Class H	Pass
**Abrasion Taber Method**	IPC SM840C Class H	Pass
**Adhesion (Copper)**<br>**(Tin / Lead)**	IPC SM840C Class H<br>IPC SM840C Class H	Pass<br>Pass
**Flammability**	UL 94V0 Rating	File No. E83564
**Ionic Contamination**	MIL-PRF-55110F	<0.3µg. NaCl/cm²<br>Using Alpha Ionograph 500M

Figure S4: Excerpt from the Technical information Leaflet of SunChemical that includes data of 'CAWN1346' and 'CAWN1274' used in this study.
